# The Missing Link: Cre Pigs for Cancer Research

**DOI:** 10.3389/fonc.2021.755746

**Published:** 2021-10-08

**Authors:** Daniela Kalla, Krzysztof Flisikowski, Kaiyuan Yang, Laura Beltran Sangüesa, Mayuko Kurome, Barbara Kessler, Valeri Zakhartchenko, Eckhard Wolf, Heiko Lickert, Dieter Saur, Angelika Schnieke, Tatiana Flisikowska

**Affiliations:** ^1^ Chair of Livestock Biotechnology, Department of Molecular Life Sciences, School of Life Sciences, Technische Universität München, Freising, Germany; ^2^ Institute of Diabetes and Regeneration Research, Helmholtz Zentrum München, Munich, Germany; ^3^ Chair of Molecular Animal Breeding and Biotechnology, Gene Center and Department of Veterinary Sciences, Ludwig-Maximilians-Universität München, Munich, Germany; ^4^ Klinik und Poliklinik für Innere Medizin II, Klinikum rechts der Isar, Technische Universität München, Munich, Germany

**Keywords:** PTF1A, iCre recombinase, pancreatic cancer, genome editing, translational model, pig, cell fate

## Abstract

The Cre/loxP system is a powerful tool for the generation of animal models with precise spatial and temporal gene expression. It has proven indispensable in the generation of cancer models with tissue specific expression of oncogenes or the inactivation of tumor suppressor genes. Consequently, Cre-transgenic mice have become an essential prerequisite in basic cancer research. While it is unlikely that pigs will ever replace mice in basic research they are already providing powerful complementary resources for translational studies. But, although conditionally targeted onco-pigs have been generated, no Cre-driver lines exist for any of the major human cancers. To model human pancreatic cancer in pigs, Cre-driver lines were generated by CRISPR/Cas9-mediated insertion of codon-improved Cre (iCre) into the porcine *PTF1A* gene, thus guaranteeing tissue and cell type specific function which was proven using dual fluorescent reporter pigs. The method used can easily be adapted for the generation of other porcine Cre-driver lines, providing a missing tool for modeling human cancers in large animals.

## Introduction

In recent decades, genetically modified mice have dominated basic and preclinical research into human cancer and many other diseases, largely because of the availability of murine ES cells and the technical ease of engineering interesting mutations. Mice are an invaluable source of knowledge but are not always the most suitable means of translating new findings into clinical application. The shortcomings of the exclusive use of rodents in preclinical studies, for example, drug trials, are now widely recognized (1) and regulatory agencies around the world are requiring preclinical trial data from non-rodent species (2). As mammals, mice and humans share many fundamental similarities, but dissimilar protein interactions, physiology, and anatomy can lead to different disease phenotypes from similar genetic lesions (3). There is therefore a strong need to investigate a wider variety of potential animal models. Pigs share many similarities with humans in body size, anatomy, and their physiological and pathophysiological responses and are playing an ever more important role in preclinical research. Many practical requirements necessary for the use of pigs as models of human cancer and cancer predisposition are in place. “Oncopigs” have been generated, carrying Cre inducible mutations in tumor suppressor (*TP53^R167H^*) or oncogenes (*KRAS^G12D^*) either as transgenes (4) or as targeted mutations of the endogenous genes (5, 6). As in mice, tumorigenesis is dependent on efficient and organ specific activation of the latent genes. But unlike for mice, no cancer specific porcine Cre-driver lines are available.

Here we report the efficient generation, tissue specificity, and *in vivo* functionality of two porcine Cre-driver lines for induction of pancreatic cancer, which has one of the highest unmet clinical needs (7, 8). Using CRISPR/Cas9 guided insertion, codon-improved Cre (iCre) was placed either at the 5’ or 3’ end of the porcine pancreas transcription factor 1 subunit alpha (*PTF1A, p48*) locus, bringing iCre expression under the control of the endogenous *PTF1A* promoter. *PTF1A* is one of the key regulators in pancreas organogenesis (9) and murine *Ptf1a-Cre* lines are commonly used for the generation of pancreatic ductal adenocarcinomas (PDAC) mouse models (10). These pig Cre lines are now available for the generation of porcine models of pancreatic cancer.

## Methods

### Constructs for Genome Editing

**Figure 1:**
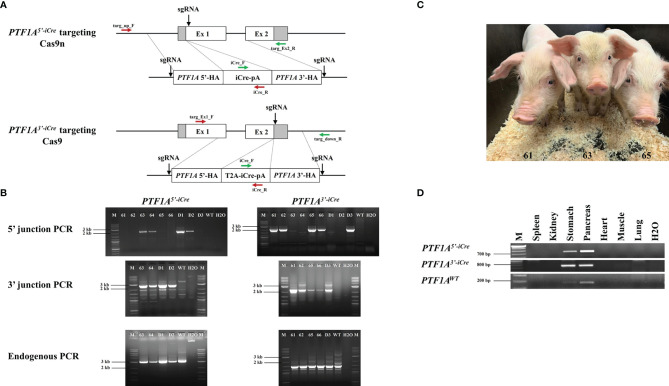
Generation of *PTF1A-iCre* founder pigs. **(A)** Genome editing strategy for iCre insertion into the 5’ or 3’end of the porcine *PTF1A* gene, using either Cas9 nickase (Cas9n) or conventional Cas9. Primer positions for targeting PCR across the 5’ and 3’ junctions are indicated as red and green arrows and for the non-targeted allele, the following primers were used: targ_up_F/targ_Ex2_R for *PTF1A^5’iCre^* and targ_Ex1_F/targ_down_R for *PTF1A^3’iCre^* pigs. **(B)** PCR analysis of 6 viable (61-66) and 3 dead (D1-D3) cloned piglets. Left panel: *PTF1A^5’iCre^* targeting PCRs. Amplified product for 5’ junction, 2233 bp; 3’ junction, 2346 bp; non-targeted allele, 3020 bp. Right panel: *PTF1A^3’iCre^* targeting PCRs. Amplified product for 5’ junction, 2127 bp; 3’ junction, 2300 bp; non-targeted allele, 2887 bp. Wild-type DNA was used as a control. M: Size marker. **(C)** Five weeks old *PTF1A^5’iCre^* (Pig_Id. 63) and *PTF1A^3’iCre^* (Pig_Ids. 61 and 65) piglets **(D)** RT-PCR analysis of *PTF1A-iCre* expression in different organs collected from the newborn founder animals.

For CRISPR/Cas9-mediated cleavage of the first exon or 3’ end of porcine *PTF1A*, the following sgRNAs were selected (5’end targeting: 5’-gtcctcttactttgacgagg-3’; 3’end targeting: 5’-acataaccagatcctcagga-3’) and cloned into a pX462-Cas9^D10A^ nickase (Cas9n; D10A variant) (5’end) or pX330-Cas9 vector (3’end) carrying a puromycin resistance. For the generation of the targeting vectors, the homology arms were amplified by PCR from genomic DNA of German landrace pigs. The 5’ targeting vector/donor DNA consists of 1053 bp 5’ and 1246 bp 3’ homology arms (HA) flanking the iCre-polyA sequence. The 3’ donor DNA consists of 900 bp 5’ and 3’ HAs, a T2A self-cleaving peptide sequence, and the iCre-polyA sequence. To increase the efficiency of homology-directed repair (HDR) and the associated knock-in of iCre, the homology arms were flanked by the respective sgRNA target sequence in both targeting vectors, leading to the generation of double cut donors (11) (**Figure 1A**).

### Cell Culture

Porcine adipose-derived mesenchymal stem cells (ADMSCs) and porcine kidney fibroblast cells (KFCs) were isolated from a male German Landrace pig by the standard method (12). ADMSCs and KFCs were cultured with high-glucose DMEM supplemented with 10% FBS, 2 mM NEAA, 2 mM L-glutamine. Cells were passaged every 3-5 days and maintained at 50-90% confluency in an incubator at 37°C and 5% CO_2_. All chemicals were purchased from Sigma-Aldrich (St. Louis, MO).

### Generation of Genome-Edited Porcine Cell Clones

Passage 0 KFCs were co-transfected with pX462-Cas9^D10A^ and 5’ donor DNA vectors for targeting into exon 1 and passage 1-2 ADMSCs with pX330-Cas9 and 3’ donor DNA vectors for iCre placement into 3’ end of one *PTF1A* allele. Transfection was performed by electroporation of 1 x 10^6^ cells using 300V and 2 pulses (BTX ECM 830, Electro Square Porator). Following plasmid concentrations were used for transfection: 5’end targeting: 1.5 µg pX462-CRISPR/Cas9n and 4 µg 5’ double cut donor DNA; 3’end: 1 µg pX330-CRISPR/Cas9 and 4 µg 3’ double cut donor DNA. One day after transfection, the cells were selected for 48 h with 1.5 µg/ml (5’end targeting) and 3 µg/ml puromycin (3’ end targeting) and then expanded and analysed as single cell clones.

### Targeting PCR

DNA isolated from individual stable transfected cell clones or founder animals was used for targeting PCR across the 5’ and 3’ junctions of the targeted *PTF1A* allele using the following primers for a) 5’end targeting: targ_up_F (5’-ctctctggagcctggctttta-3’) and iCre_R (5’-agagtcatccttggcaccat-3’) for 5’ junction PCR; iCre_F (5’-caagctggtggagagatgga-3’) and targ_Ex2_R (5’-ctggccagagttgttccaac-3’) for 3’ junction PCR (**Figure 1A**). The amplified PCR products were 2233 bp for 5’ and 2346 bp for 3’ junction respectively (**Figure 1B**). b) 3’end targeting: targ_Ex1_F (5’-ctgcacgagtactgctaccg-3’) and iCre_R for 5’ junction PCR and iCre_F and targ_down_R (5’-gctgaaagggatgagagggt-3’) for 3’ junction PCR (**Figure 1A**). The PCR products were 2127 bp for 5’ and 2300 bp for 3’ junction PCR respectively (**Figure 1B**). Correct insertion was confirmed by DNA sequencing of the PCR products.

Detection of a nontargeted *PTF1A* wild-type allele confirmed the monoallelic iCre insertion. For PCR amplification of the wild-type allele the following primers were used: targ_up_F and targ_Ex2_R for *PTF1A^5’-iCre^* and targ_Ex1_F and targ_down_R for *PTF1A^3’-iCre^* (**Figure 1A**), resulting in PCR products of 3020 bp and 2887 bp, respectively (**Figure 1B**). All PCRs were carried out with Phire Hot Start II DNA Polymerase (Thermo Fisher Scientific). Cycling thermal conditions were: 4 min, 98°C then 40 cycles of: 20 s, 98°C, 20 s, 65°C (for 5’ junction PCR), 63.6°C (3’ junction PCR), 64°C (for 5’ and 3’ endogenous PCR), 55 s, 72°C followed by 3 min 72°C.

### RT-PCR

Total RNA was isolated using InnuSPEED Tissue RNA Kit (Analytic Jena). After DNase digest (Turbo DNA-free™ Kit, Invitrogen), RNA was reverse transcribed using FastGene Scriptase II cDNA Kit (Nippon Genetics). RT-PCR was performed with Phire Hot Start II DNA Polymerase (Thermo Fisher Scientific) using forward primer hybridizing to exon 1 of *PTF1A* (*PTF1A^5’-iCre^*: 5’-ctctcagcttcagcacatcg-3’, *PTF1A^3’-iCre^*: 5’-gaaggtcatcatctgccacc-3’) and reverse primer to the iCre (5’-ggtcaaagtcagtgcgttca-3’), resulting in PCR products of 701 bp and 746 bp in length. Wildtype *PTF1A* mRNA expression was examined using primers hybridizing to exon 1 and 2 of *PTF1A* (5’-gaaggtcatcatctgccacc-3’ and 5’-ttgagtttcctggggtcctc-3’), resulting in a PCR product of 174 bp in length.

### Off-Target Analysis

Off-targets sites for sgRNA for 5’ and 3’end targeting strategy were predicted with the use of CRISPOR (www.crispor.tefor.net) website tool. Five highest scoring potential off-target sites were analysed by PCR followed by sequncing.

### Somatic Cell Nuclear Transfer (SCNT)

Two selected cell clones for *PTF1A^5’iCre^* targeting and three cell clones for *PTF1A^3’iCre^* targeting were sent for SCNT. SCNT was performed as previously described (13).

### Immunohistochemistry (IHC) Staining

Specimens were fixed in 4% formaldehyde solution for 48 h, embedded in paraffin, and sectioned (2 µm). After deparaffinization, antigen demasking (citrate buffer, pH=6) and inactivation of endogenous peroxidases (3% H_2_O_2_ in methanol), permeabilization was performed (1% goat serum, 0.05% Tween-20 and 0.4% Triton-X-100 in PBS) for 10 min. After blocking for 1 h (2% goat serum in PBS), sections were incubated overnight at 4°C with primary antibodies. Following antibodies were used: anti-Cre Recombinase in 1:100 dilution (D7L7L XP^®^ Rabbit mAb #15036, Cell Signaling). After 1 h incubation with Horseradish peroxidase (HRP)-conjugated secondary antibody (Goat Anti-Rabbit IgG-HRP #4030-05, SouthernBiotech) in 1:200 dilution, detection was performed using Vector DAB substrate (Vector Laboratories). Afterwards, the sections were counterstained with hematoxylin.

### Immunofluorescence (IF) Staining

The pancreata were dissected, embedded in tissue freezing medium (Leica Biosystems), and sectioned at 12 μm. Tissue sections were fixed in pre-chilled 4% paraformaldehyde for 15 min at 4°C, then dehydrated in a progressive ethanol gradient at room temperature. Sections were permeabilized for 30 min at 40°C, blocked (10% FCS, 0.1% BSA, and 3% donkey serum in PBS with 0.1% Tween-20) for 1 h at room temperature (RT), and incubated with primary antibodies overnight at 4°C. Sections were washed in PBS, incubated with secondary antibodies (1:800 in blocking solution) for 3 h at 4°C, counterstained with DAPI for 20 min at RT, and mounted with the ProLong™ Diamond Antifade Mountant (ThermoFisher). Slides were dried overnight at RT and stored at 4°C until imaging by confocal microscope (ZEISS, LSM 880 with Airyscan). The following primary antibodies were used for immunofluorescent stainings: chicken anti-GFP (1:100, ThermoFisher); rat anti-RFP(1:500, Chromotek); rabbit anti-RFP (1:500, Rockland); rabbit anti-cytokeratin (1:500, Agilent); mouse anti-insulin (1:1000, Sigma); mouse anti-glucagon (1:500, Sigma); mouse anti-CPA1 (1:1000, LSBio). The following secondary antibodies were used: donkey anti-chicken Alexa Fluor 488 (Dianova); donkey anti-rat Cy3 (Dianova); donkey anti-rabbit Alexa Fluor 555 (ThermoFisher); donkey anti-mouse Cy5 (Dianova); donkey anti-rabbit AlexaFluor 649 (ThermoFisher).

## Results

### Generation of PTF1A^5’iCre^ and PTF1A^3’iCre^ Pigs

#### PTF1A^5’iCre^

In the *Ptf1a-Cre* mouse line the Cre recombinase gene had been inserted into exon 1 downstream to the translational start codon, silencing the expression of the endogenous gene. As the basic helix-loop-helix transcription factor PTF1A/p48 is critical for pancreatic cell fate it is essential that only one allele is targeted. To replicate the murine model targeted gene placement supported by CRISPR/Cas9 was carried out in primary porcine cells isolated from a male German Landrace pig. To ensure that only one allele was targeted a Cas9 nickase (Cas9n; D10A variant) was used (14). To improve the efficiency of HDR, both 5’ and 3’ short regions of homology (approx. 1 kb) of the iCre (codon improved Cre)-donor plasmid were flanked by two sgRNA target sequences, which were identical to the target sequence in exon 1 of *PTF1A* (**Figure 1A**) (11). The expression vector for both Cas9n and the exon1 sgRNA was co-transfected with the iCre double cut donor DNA plasmid. Single cell clones were isolated and screened by 5’- and 3’-junction PCR for correct gene targeting (**Figure 1B**). HDR efficiency was 25%.

#### PTF1A^3’iCre^

PTF1A/p48 is crucial for establishing the differentiation state of acinar cells. In mice, *Ptf1a* loss in adult acinar cells causes ER stress and activates the apoptosis pathways (15). It is epigenetically downregulated in pancreatic ductal adenocarcinoma (PDAC) and is assumed to be a tumor suppressor gene (16). As it was unknown if a heterozygous inactivation of *PTF1A* would have any adverse effects on porcine organogenesis or influence PDAC tumorigenesis, an alternative approach where iCre was placed at the 3’end of *PTF1A*, has been used. The addition of a “self-cleaving” 2A peptide (17) should allow translation of both *PTF1A* and *iCre* from a single mRNA. Again, a double cut iCre donor vector was generated (**Figure 1A**), genome editing was carried out as described above using Cas9 instead of Cas9n vector giving HDR efficiency of 11%.

For both gene editing strategies the five highest scoring potential off-target sites were determined by the CRISPOR web tool (18). PCR amplification across these sites followed by sequence analyses showed no off-target events **(Supplementary Figure 1**).

Five correctly targeted primary cell clones for both *PTF1A^5’iCre^* and *PTF1A^3’iCre^* were pooled and used for SCNT. Nine male piglets were born: four *PTF1A^5’iCre^* and five *PTF1A^3’iCre^* (**Figure 1C**). Three piglets (two *PTF1A^5’iCre^* and one *PTF1A^3’iCre^*) died shortly after birth. The remaining six piglets were healthy and fertile. Precise iCre transgene placement into *PTF1A* allele was confirmed by 5’ and 3’ junction PCR followed by sequence analysis for all *PTF1A^5’iCre^* and *PTF1A^3’iCre^* founder animals (**Figure 1B**). In each case the presence of an untargeted wildtype allele confirmed monoallelic insertion of iCre at the *PTF1A* locus (**Figure 1B**).

### Tissue Specific Expression of Porcine PTF1A-iCre

**Figure 2:**
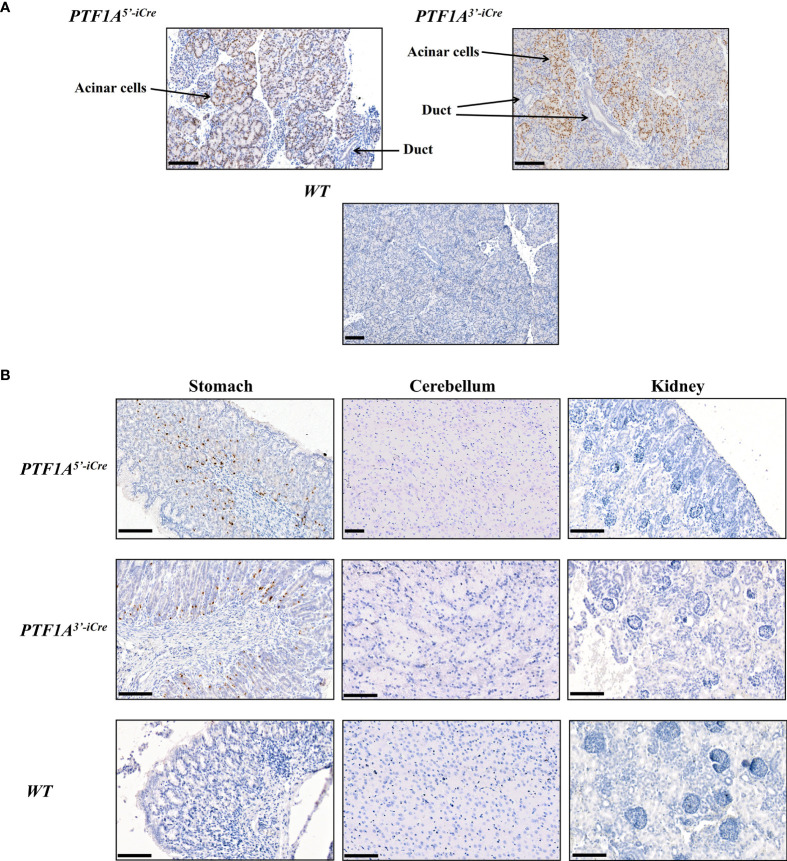
Cre protein expression in *PTF1A-iCre* animals. **(A)** IHC stained pancreata from 1-day old *PTF1A^5’iCre^* and *PTF1A^3’iCre^* piglets show iCre protein expression in pancreatic acinar cells, but not in duct cells. **(B)** IHC staining of stomach, cerebellum and kidney reveals iCre protein expression in single cells of the stomach, but no expression in brain and kidney. Scale bar represents 100 µm.

To assess *PTF1A-iCre* expression in the newborn founder animals, tissue samples (pancreas, stomach, kidney, spleen, heart, muscle and lung) were collected for RT-PCR and immunohistochemical analyses. At this developmental stage iCre expression was restricted to pancreatic acinar and single gastric cells in both *PTF1A^5’iCre^* and *PTF1A^3’iCre^* targeted pigs (**Figures 1D**, **2A, B**). The other organs tested were negative. These results are in accordance with *PTF1A/p48* expression in humans (https://www.proteinatlas.org/ENSG00000168267-PTF1A/tissue).

### PTF1A-iCre Driver Pig Lines Support Tissue Specific Cre Mediated Recombination *In Vivo*

**Figure 3:**
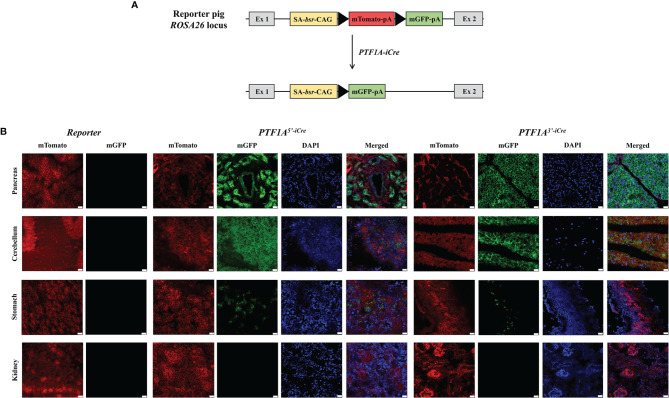
Functional analysis of PTF1A-iCre using dual fluorescent reporter pigs. **(A)** Schematic representation of the *R26^mT/mG^* before and after Cre-recombination. **(B)** mTomato/mGFP fluorescence in tissues collected from *R26^mT/mG^*/*PTF1A^5’iCre^* or *PTF1A^3’iCre^* piglets. Scale bar represents 20 µm. Ex, exon; SA, splice acceptor; bsr, blasticidin resistance gene; CAG, CAG promoter; triangle, loxP site; pA, polyA tail.

To assess the location, pattern, and extent of iCre expression during fetal development and to prove iCre function, founder boars were crossed with a dual-fluorescence reporter pig line *R26^mT/mG^* (19). This line carries a membrane-targeted tandem dimer GFP (mGFP) gene downstream of a floxed mTomato transgene at the porcine *ROSA26* locus (**Figure 3A**). Cre mediated excision of mTomato results in a switch from red to green fluorescence (20), marking all cells during development in which Cre recombinase was active.

F1 animals showed near Mendelian inheritance: 3/8 (37.5%) for *PTF1A^5’iCre^* and 4/11 (36.7%) for *PTF1A^3’iCre^*. On postnatal day 1, one *R26^mT/mG^PTF1A^5’iCre^* and one *R26^mT/mG^PTF1A^3’iCre^* piglet were sacrificed, organs isolated, and fluorescent imaging carried out. **Figure 3B** shows extensive green fluorescence in the pancreas and cerebellum and single positive cells in the stomach. Other tissues such as the kidney were negative.

**Figure 4:**
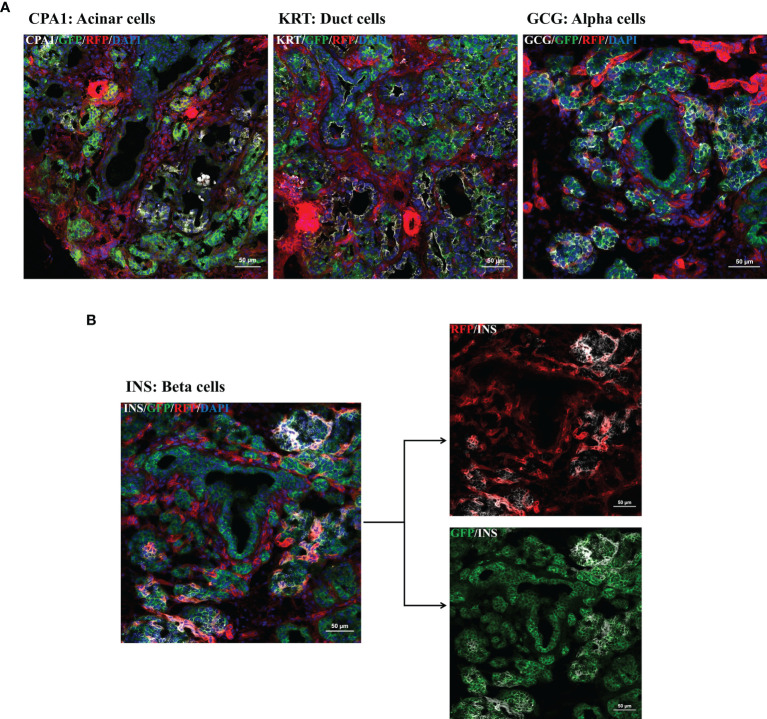
Multi-color immunofluorescence staining for pancreatic cell type determination. IF staining for pancreatic progenitors was performed using frozen tissue samples of day 1 old *R26^mT/mG^*/*PTF1A^5’iCre^* or *PTF1A^3’iCre^* piglets. **(A, B)**: Ductal cells, acinar cells, α-cells, and β-cells were stained using antibodies against cytokeratin, carboxypeptidase A, glucagon and insulin. **(B)** Co-staining of insulin with RFP and GFP. Scale bar represents 50 µm.

Focusing on the pancreas multi-color immunofluorescence staining was performed for cell type identification. As shown in **Figure 4A** mGFP is expressed in glucagon-producing α-cells, ductal cells, and acinar cells. Interestingly, for β-cells co-staining of insulin with both RFP and GFP was observed (**Figure 4B**). Colocalization of these pancreatic cell types with GFP confirmed the participation of PTF1A to all pancreatic lineages during development.

## Discussion

Cre/loxP, Flp/FRT, and Dre/Rox are site-specific recombinase systems, which can be employed to activate or inactivate conditionally targeted genes in a tissue specific manner (21). The Cre/loxP system has been extensively used in mice for lineage tracing, to study gene and cell function, induction, and development of cancer and a large number of Cre-driver lines are available (22). This is in strong contrast to livestock species where very few Cre transgenic animals have been generated, based solely on random integration into the genome, showing expression but not *in vivo* functionality (23, 24). In the two pig lines reported here, the Cre recombinase is under the control of an endogenous promoter ensuring a correct spatial and temporal expression pattern, and *in vivo* Cre mediated recombination was confirmed.

Until recently targeted gene placement has been technically difficult in livestock species due to the lack of functional pluripotent stem cells. Gene targeting experiments were restricted to somatic cells, where homologous recombination is 100-fold less efficient. Targeting of non-expressed genes, such as pancreas specific genes, in porcine fibroblasts only became realistic with the advent of genome editing. Here we show that a method previously employed for precise knock-ins in induced pluripotent stem cells (11) allows highly efficient and precise gene placement (up to 25%) in somatic cells, which remain nuclear transfer competent. This approach is generally applicable and will enable the efficient generation of other Cre driver lines.

The functionality of *PTF1A-iCre* was confirmed by crossbreeding founder animals with our dual fluorescent reporter pig line (19). This not only proved the efficient removal of the floxed mTomato gene in the pancreas but also indicated which pancreatic cell types were derived from early *PTF1A* expressing progenitor cells, including acinar and ductal cells, α-cells but not all β-cells. After birth *PTF1A-iCre* expression became restricted to acinar cells. This is consistent with findings in mice, where *Ptf1a* was shown to be essential for commitment to pancreatic fate (25) and the formation of pancreatic multipotent progenitor cells (26). Later in development, it is expressed in acinar cells where it maintains acinar cell identity (27). Very localized *PTF1A* expression was found in the stomach both in the embryo and after birth. Gastric and pancreatic developmental programs are related, and low-level expression has also been observed in human stomach samples. During fetal development, PTF1A/p48 is also expressed in the cerebellar ventricular zone where it is required for the development of cerebellar GABAergic neurons (28). Accordingly, widespread expression of green fluorescence protein was observed in the cerebellum of *R26^mT/mG+/-^PTF1A-iCre* pigs.

Based on its importance during pancreas development and cerebellar neurogenesis, mutations in *PTF1A* can cause cerebellar ataxia and permanent neonatal diabetes mellitus in humans (29). With regard to pancreatic cancer, deletion of *Ptf1a* causes acinar to ductal metaplasia and dramatically enhances KRAS-driven acinar cell transformation in mice and downregulation is observed in human PDAC (16), leading to the postulation that *PTF1A*/*p48* functions as a tumor suppressor gene. By placing iCre at the 5’ end of the porcine *PTF1A* gene we replicated existing mouse models and have inactivated one *PTF1A* allele. By introducing iCre at the 3’ end, PTF1A/p48 protein expression should not be affected. It will be interesting to see if this alters tumorigenesis. Breeding is underway to obtain triple modified pigs (KPC pigs) in which *KRAS^G12D^* and *TP53^R167H^* are activated in a tissue specific manner by either *PTF1A^5’iCre^* or *PTF1A^3’iCre^* and -as in KPC mice- initiates PDAC as the main pathophenotype. The KPC mice showed no phenotype outside the pancreas, no gastric or neuronal cancers during their lifetime despite high PTF1A expression in the cerebellum, suggesting a tissue-specific and context-dependent response (30). While unlikely, it remains to be seen if this will be different for the pig KPC model.

Pancreatic cancer is one of the most lethal forms of cancer, with fewer than 20% of people surviving for longer than twelve months after diagnosis. The usefulness of large animal models for translational research and as pre-clinical models has been documented (31–35). Modelling PDAC in large animals was hampered by the lack of Cre-driver lines. Besides fulfilling this essential requirement for a porcine PDAC model, the technology employed enables the efficient generation of other Cre transgenic lines for cancer research. In combination with reporter pigs, these Cre-driver lines can also support genetic fate mapping.

## Data Availability Statement

The original contributions presented in the study are included in the article/**Supplementary Material**. Further inquiries can be directed to the corresponding author.

## Ethics Statement

All animal experiments were approved by the Government of Upper Bavaria (permit number ROB-55.2-2532.Vet_02-18-33).

## Author Contributions

DK, TF, HL, DS, and AS conceived the study. DK generated DNA constructs, performed transfections, and analyzed the cell clones. MK, BK, VZ, and EW performed SCNT and embryo transfer. KF, TF, and DK collected samples. DK performed molecular analysis of the PTF1A-iCre founder animals. DK, TF, CY, and LB performed histological analysis. DK wrote the manuscript. KF, TF, and AS edited the manuscript. All authors critically reviewed and approved the final manuscript.

## Funding

This work was supported by: the Deutsche Forschungsgemeinschaft (DFG, German Research Foundation) SFB1321: Project ID 329628492 and European Union's Horizon 2020: PAVE (GA 861190).

## Conflict of Interest

The authors declare that the research was conducted in the absence of any commercial or financial relationships that could be construed as a potential conflict of interest.

## Publisher’s Note

All claims expressed in this article are solely those of the authors and do not necessarily represent those of their affiliated organizations, or those of the publisher, the editors and the reviewers. Any product that may be evaluated in this article, or claim that may be made by its manufacturer, is not guaranteed or endorsed by the publisher.
