# Supplementary material for: The Missing Link: Cre Pigs for Cancer Research
Source: Front Oncol. 2021 Oct 8;11:755746. doi: 10.3389/fonc.2021.755746 (PMC8531543; doi:10.3389/fonc.2021.755746)
Supplement: Supplementary file 1 [file DataSheet_1.pdf]

A)

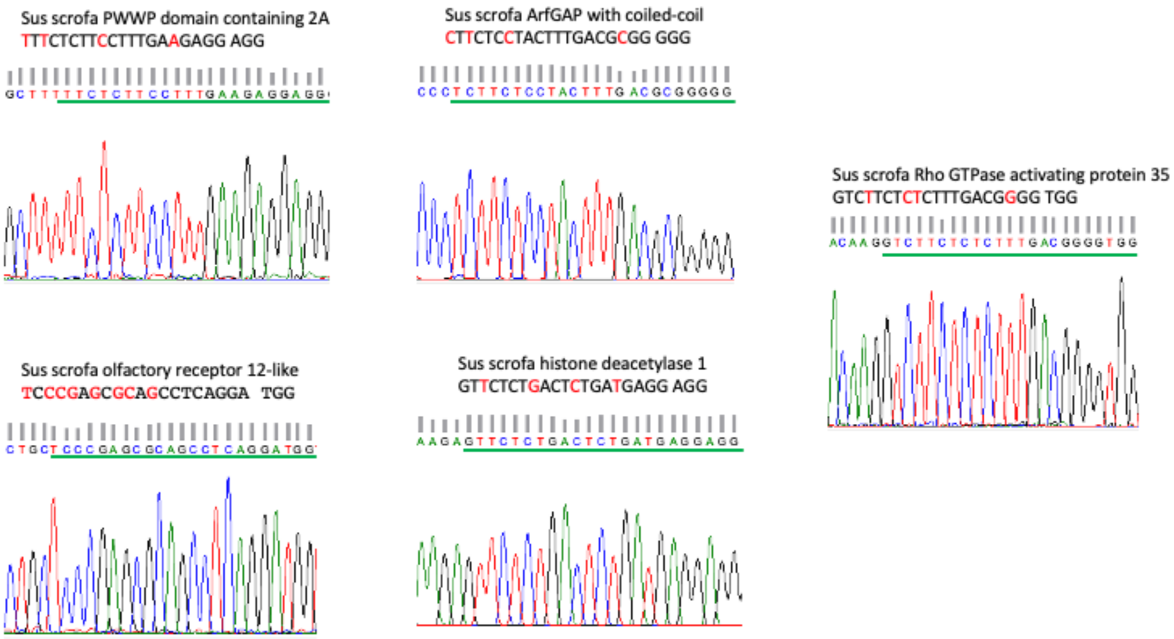

B)

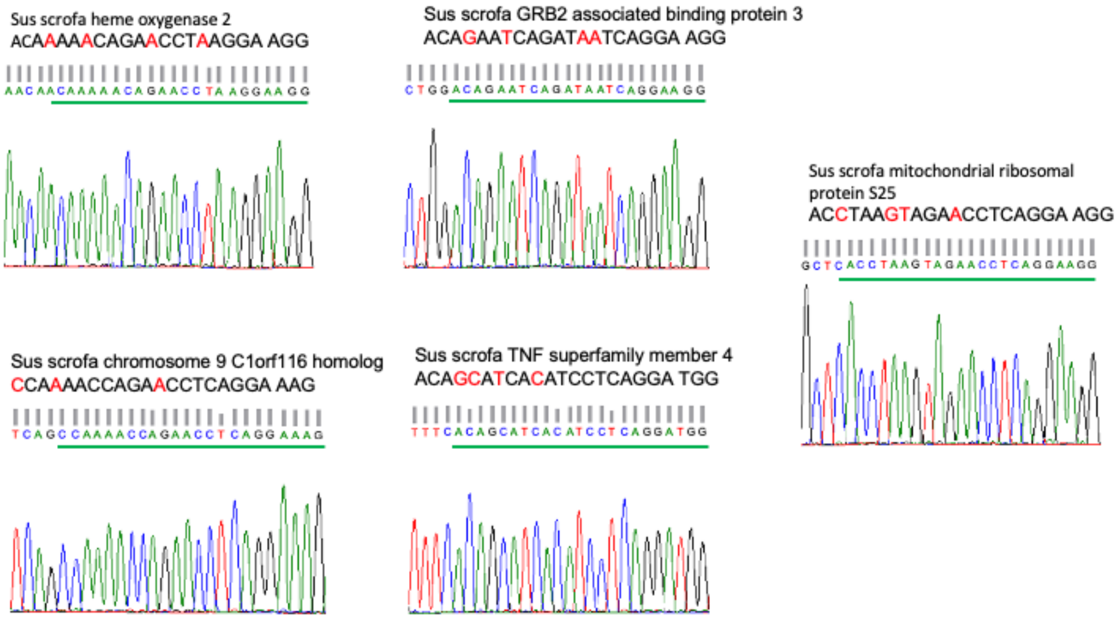

Supplementary Figure 1. Sequence analysis of five potential off-targets for *PTF1A* (A): 5'end gRNA, (B): 3'end gRNA. The mismatches to on-target sites are marked in red. The potential off-target sites (including PAM sequence) are underlined.
